# Probing Molecular Motions in Metal–Organic Frameworks by Three-Dimensional Electron Diffraction

**DOI:** 10.1021/jacs.1c08354

**Published:** 2021-10-25

**Authors:** Laura Samperisi, Aleksander Jaworski, Gurpreet Kaur, Karl Petter Lillerud, Xiaodong Zou, Zhehao Huang

**Affiliations:** † Department of Materials and Environmental Chemistry, Stockholm University, Stockholm SE-106 91, Sweden; ‡ Department of Organic Chemistry, Stockholm University, Stockholm SE-106 91, Sweden; § Department of Chemistry, Center for Materials Science and Nanotechnology, University of Oslo, P.O. Box 1033, N-0315 Oslo, Norway

## Abstract

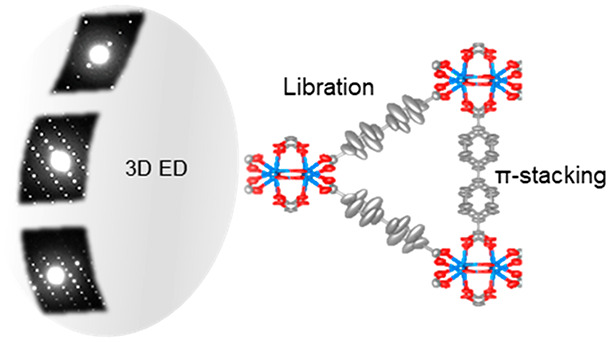

Flexible metal–organic frameworks (MOFs) are known for their vast functional diversities and variable pore architectures. Dynamic motions or perturbations are among the highly desired flexibilities, which are key to guest diffusion processes. Therefore, probing such motions, especially at an atomic level, is crucial for revealing the unique properties and identifying the applications of MOFs. Nuclear magnetic resonance (NMR) and single-crystal X-ray diffraction (SCXRD) are the most important techniques to characterize molecular motions but require pure samples or large single crystals (>5 × 5 × 5 μm^3^), which are often inaccessible for MOF synthesis. Recent developments of three-dimensional electron diffraction (3D ED) have pushed the limits of single-crystal structural analysis. Accurate atomic information can be obtained by 3D ED from nanometer- and submicrometer-sized crystals and samples containing multiple phases. Here, we report the study of molecular motions by using the 3D ED method in MIL-140C and UiO-67, which are obtained as nanosized crystals coexisting in a mixture. In addition to an *ab initio* determination of their framework structures, we discovered that motions of the linker molecules could be revealed by observing the thermal ellipsoid models and analyzing the atomic anisotropic displacement parameters (ADPs) at room temperature (298 K) and cryogenic temperature (98 K). Interestingly, despite the same type of linker molecule occupying two symmetry-independent positions in MIL-140C, we observed significantly larger motions for the isolated linkers in comparison to those reinforced by π–π stacking. With an accuracy comparable to that of SCXRD, we show for the first time that 3D ED can be a powerful tool to investigate dynamics at an atomic level, which is particularly beneficial for nanocrystalline materials and/or phase mixtures.

Structural flexibility is a unique characteristic of metal–organic frameworks (MOFs)/porous coordination polymers (PCPs).^1−6^ It plays a central role in the host–guest chemistry and is attractive for a wide range of applications, including gas storage,^7−10^ catalysis,^11−13^ and separation.^14−17^ In addition to the well-known phenomenon of breathing and swelling, local flexibilities, such as linker rotation and swing, are not accompanied by phase transitions yet play an important role in controlling the pore accessibility and diffusion rates of the guest molecules in the framework.^18−21^ As the dynamic properties are closely associated with structural features, a deep understanding of MOFs, down to an atomic level, is essential for further development of rational design strategies and exploitation of their applications.

In MOF synthesis, it is often found that the same starting regents (i.e., metals and organic ligands) can lead to MOFs with different topologies, which are dictated by experimental thermodynamic and/or kinetic conditions.^22,23^ The coexistence of different MOFs in the same sample hampers characterization by many techniques, such as nuclear magnetic resonance (NMR) and powder X-ray diffraction (PXRD), to unambiguously determine the structure–property relationship. Additionally, MOFs are often only obtained as nanosized crystals, which are too small to be studied by single-crystal X-ray diffraction (SCXRD). Transmission electron microscopy (TEM) provides unique advantages in investigating MOFs at an atomic level, even when they appear as a phase mixture. With the recent development of real-space TEM imaging techniques, direct observation of MOFs has been achieved at atomic resolution, which revealed changes during MOF formation, phase transition, and host–guest interactions.^24,25^ While TEM imaging is advantageous for analyzing local structures, it can only be applied to limited MOFs that can stand relatively high electron doses. In comparison to imaging, three-dimensional electron diffraction (3D ED) requires a 2 magnitudes lower electron dose,^24^ with a dose rate of approximately 0.01 e^–^ A^–2^ s^–1^. The strong interaction between electrons and matter allows single-crystal 3D ED studies on nanometer-sized MOFs.^26−34^ 3D ED thus offers an immerse opportunity to determine and refine 3D structures of beam-sensitive nanomaterials.^35−38^

Linker motions in MOFs (e.g., π-flips and small-angle librations) are commonly detected by solid-state NMR,^39,40^ vibrational spectroscopy,^41^ and inelastic neutron scattering.^42^ Whereas SCXRD has also been used to elucidate dynamics in crystalline solids,^43−45^ such studies have not been demonstrated by electron diffraction. Here, we present the first study of molecular motions in MOFs by 3D ED using the data collection protocol of continuous rotation electron diffraction (cRED) developed in our group.^46^ Using Zr as the metal source and 4,4′-biphenyldicarboxylate (bpdc) as the organic linker, UiO-67 and MIL-140C were synthesized in a phase mixture, with a ZrCl_4_:H_2_bpdc:benzoic acid:*N*,*N*-dimethylformamide (DMF):water molar ratio of 1:1:3:50:3 (Figures S1 and S2; see the Supporting Information for more details). This condition has been reported to synthesize UiO-67 with little missing-linker defects.^47^ This phase mixture was used for the current study, and 3D ED has the unique advantage of studying multiphasic samples.^33,48^

The cRED data collected from the sample gave two distinct unit cells and space groups: one with the cubic space group *Fm*3̅*m* and *a* = 26.880(3) Å corresponding to UiO-67^49^ (Figure S3) and the other with the monoclinic space group *C*2/*c* and *a* = 32.360(7) Å, *b* = 15.800(3) Å, *c* = 7.910(2) Å, and β = 103.00(3)°, attributed to MIL-140C^50^ (Figure S4). Details of data collection and space group determination are given in the Supporting Information. The structures of both MOFs were solved *ab initio* from cRED data using a single data set for UiO-67 and a merged data set from three different crystals for MIL-140C. All non-hydrogen atoms were found directly from the structure solution and refined with anisotropic atomic displacement parameters. The refinements converged to a R1 value of 0.215 for 404 observed reflections for UiO-67 and 0.169 for 2083 observed reflections for MIL-140C (Table S1).

UiO-67 is built by one 12-connected Zr_6_O_4_(OH)_4_ cluster and one bpdc linker in the asymmetric unit, and its structure was previously determined by SCXRD.^51^ The structural model refined against the cRED data shows severe elongations of the nonaxial carbon atoms of the bpdc linker (C3 and C4) in the thermal ellipsoid model (Figure 1a). As thermal ellipsoids represent the probability of atomic positions,^52^ the elongations could be attributed to molecular motions of the bpdc linker. This observation is in good agreement with that simulated by molecular dynamics (Figure S5)^53^ and obtained by SCXRD. Despite the fact that the cRED data have a much lower resolution (0.90 Å) in comparison to the SCXRD data (0.70 Å), the ellipsoids from cRED data have a comparable spatial coverage with the split atoms of the SCXRD model (Figure 1b).

**Figure 1:**
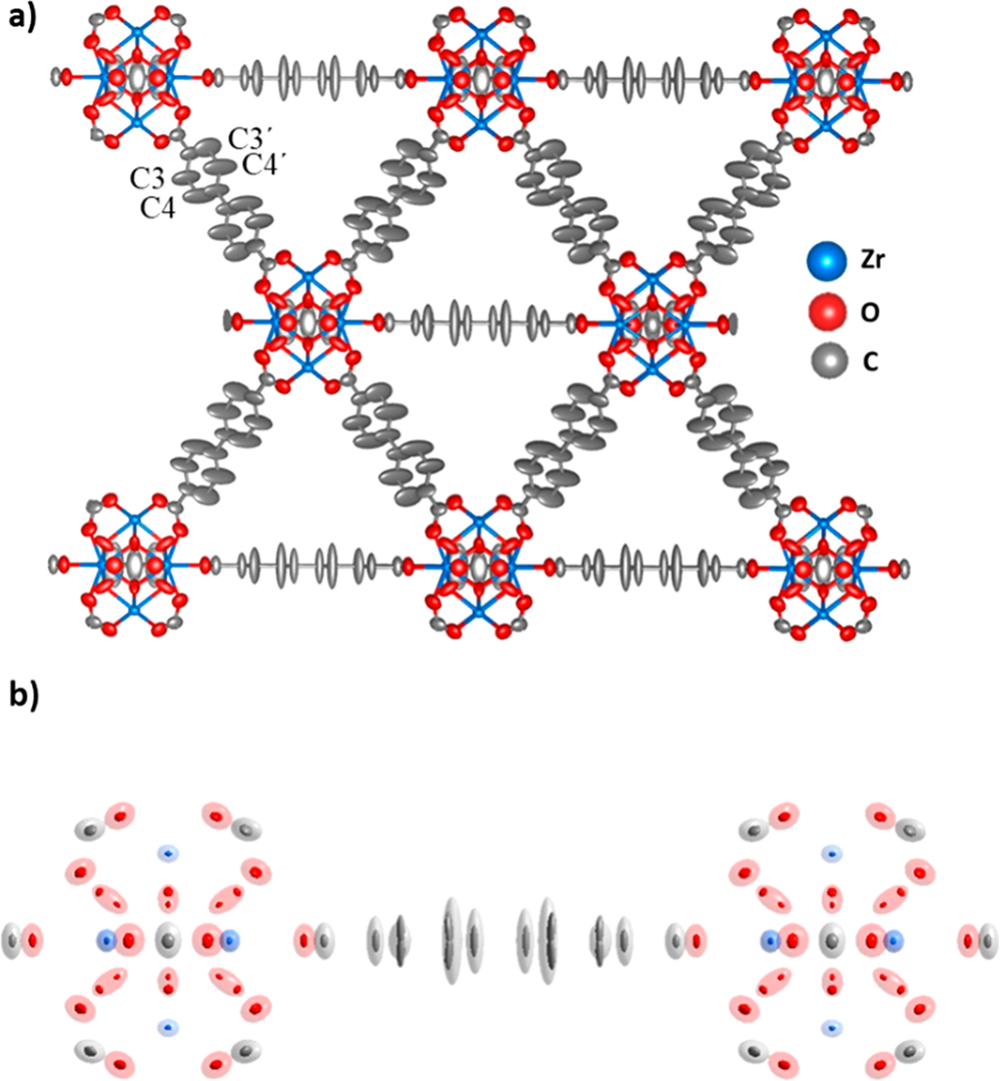
(a) Thermal ellipsoid model (50% probability) of UiO-67 obtained after refinement against cRED data. (b) Superimposed thermal ellipsoid models of UiO-67 obtained from cRED (transparent) and SCXRD (solid) data. Atomic bonds are removed for clarity.

In addition to the molecular motions, we also found an unusual elliptical behavior of the μ_3_-O atom caused by the disorder in the Zr_6_ cluster, which was also observed by SCXRD.^51^ For the other atoms in the structure, the atomic positions of UiO-67 obtained by cRED and SCXRD exhibited an excellent agreement, with an average deviation of 0.018(1) Å (Table S2).

The structure of MIL-140C was previously reported by model building through reticular chemistry,^50^ and its single-crystal analysis has not been achieved until this work. MIL-140C is built by Zr-oxo chains rather than Zr-oxo clusters. There are two bpdc linkers in the asymmetric unit. The Zr-oxo chains are aligned in parallel to [001] and are connected by linker 1 along the [110] and [1–10] directions and by linker 2 along [010]. The thermal ellipsoid model obtained from cRED data shows strong elongations of the nonaxial carbon atoms in linker 1 (Figure 2a). In contrast, the elongations are much smaller for the atoms in linker 2. The electrostatic potential map further confirms the difference in the shape of the potential peaks between the nonaxial carbon atoms in linkers 1 and 2 (Figure 2b). The nonaxial carbon peaks of linker 2 have well-defined positions in comparison to those of linker 1, indicating that the atoms in linker 2 are more ordered and have much less motion.

**Figure 2:**
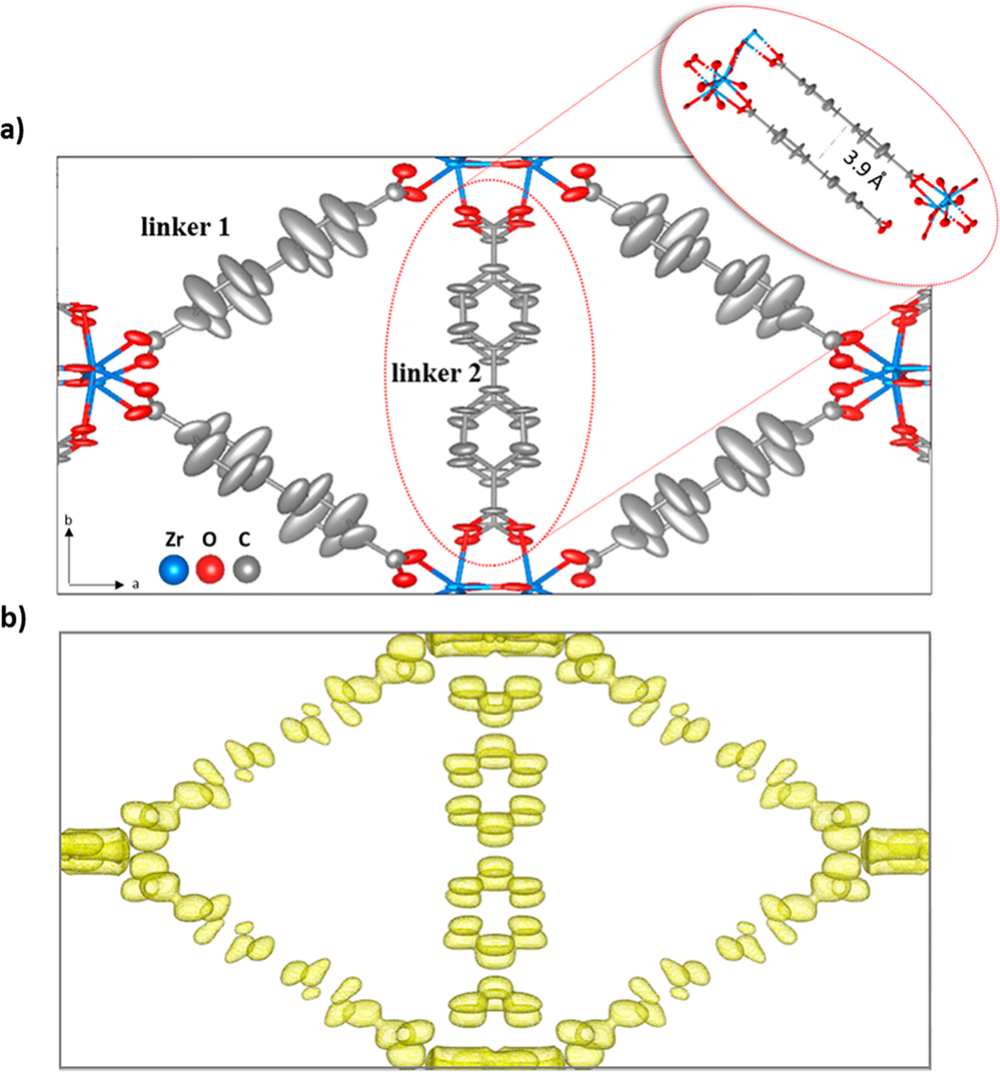
(a) Thermal ellipsoid model (50% probability) of MIL-140C obtained after refinement against cRED data. The model is viewed along the [001] direction. (b) The electrostatic potential map, which was contoured at the 2σ level.

The ellipsoids of linker 1 elongate perpendicularly to their linker planes. Within the MOF structure, linkers 1 and 2 have different chemical environments. For linker 2, the distance between a pair of its linker planes is 3.9 Å. This indicates a weak π–π interaction, which stabilizes the molecules. As a result, linker 2 molecules are confined in their position, resulting in smaller ellipsoids and more localized potential peaks. In contrast, linker 1 does not form any interaction with other linkers and its large spatial freedom could give raise to molecular motions. Indeed, its stretched ellipsoids resemble the dynamic behavior of the bpdc molecules in UiO-67.

As thermal ellipsoids are an expression of atomic anisotropic displacement parameters (ADPs), we further explore the ADPs obtained by cRED data to better understand the structural details of MIL-140C. We collected data at room temperature (298 K) and cryogenic temperature (98 K), and investigated the behaviors of the ADPs. At each temperature, nine individual data sets were collected from different crystals, which were combined to generate three merged data sets, each from three individual data sets (Tables S3 and S4). We performed anisotropic structure refinement against each of the merged data sets of MIL-140C and plotted the three eigenvalues (λ1, λ2, and λ3) of the four nonaxial carbon atoms of each linker calculated from the corresponding ADPs,^54^ as shown in Figure 3c. The eigenvalues describe the mean-square displacements along the three axes of the ellipsoids, with reduced eigenvalues implying smaller atomic displacement.^43^ While no significant changes are observed on the eigenvalues at different temperatures, λ1 values of the nonaxial carbon atoms in both linkers are larger than λ2 and λ3 values (Figure 3). While the λ2 and λ3 values are similar among all the nonaxial carbon atoms in linkers 1 and 2, the λ1 values are more than twice as small in linker 2 as those in linker 1. This confirms the rigid behavior of linker 2. It should be noted that many factors, such as incomplete data and dynamic effects, can introduce artifacts on ADPs. However, despite the missing cone due to the preferred crystal orientation of MIL-140C (Figure S6), only the nonaxial carbons in linker 1 exhibit severe elongation of the ellipsoids. This indicates that molecular motion is the predominant factor that causes the elongation. The spatial freedom surrounding linker 1 could facilitate its motion as either small-amplitude librations or π-flips (Figure S7) around the molecular axis.

**Figure 3:**
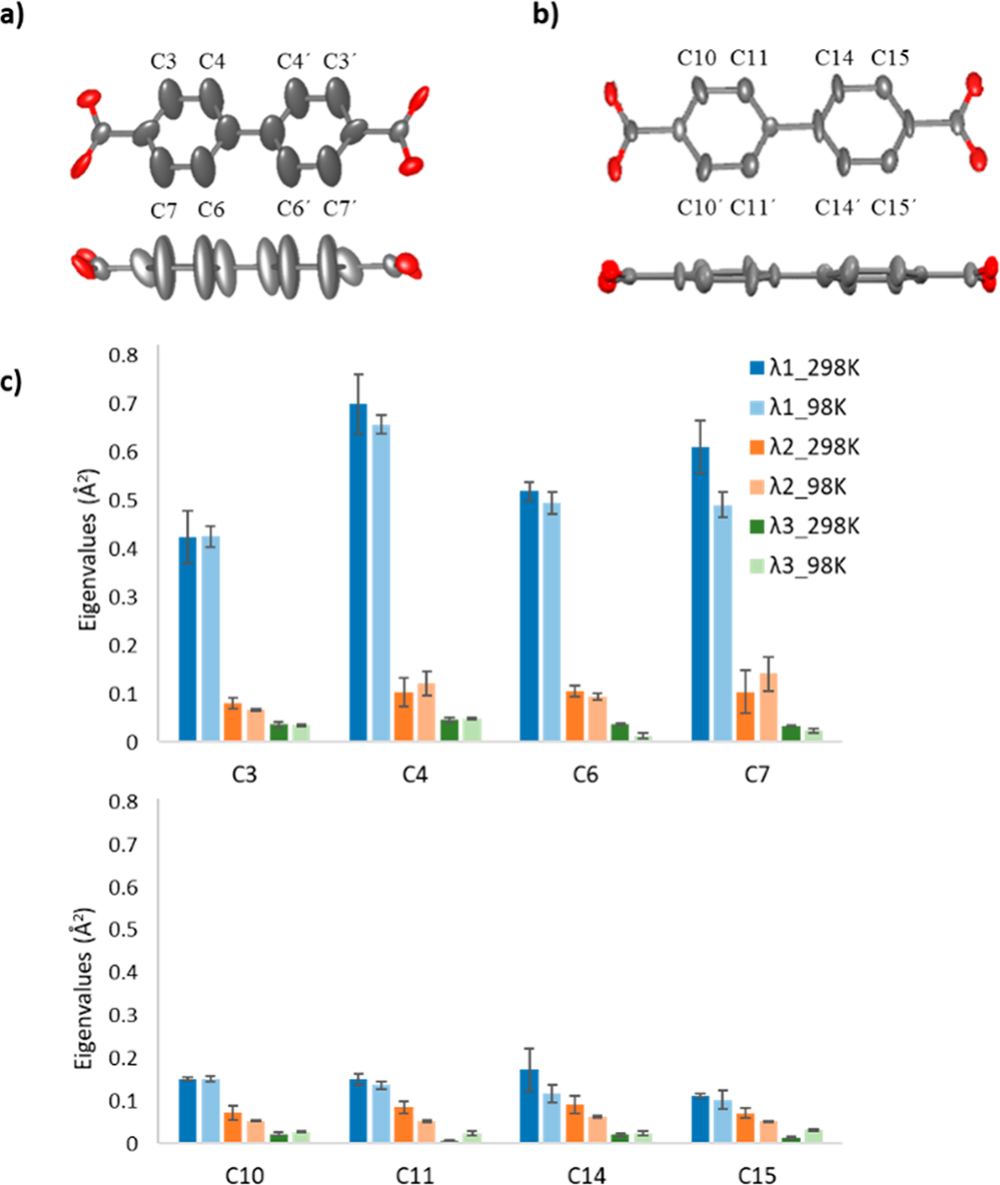
Thermal ellipsoid model (50% probability) of (a) linker 1 and (b) linker 2 in MIL-140C. (c) Eigenvalues (λ1, λ2, and λ3) at 298 and 98 K calculated from the ADPs of the nonaxial carbon atoms in linker 1 (C3, C4, C6, and C7) and linker 2 (C10, C11, C14, and C15).

π-flips are fast high-amplitude motions that occur only when relatively large energy barriers are overcome.^39,55^ Solid-state NMR on pure MIL-140C (see the Supporting Information for synthesis details) was applied to further confirm the type of rotational motion of linker 1. The ^1^H–^13^C CPMAS spectrum at a slow MAS rate of 7 kHz was recorded to allow observation of the ^13^C chemical shift anisotropy (CSA) pattern (Figure S8). The estimation of the chemical shift anisotropy (δ_aniso_) as well as the shape of the CSA pattern provides insight into the potential motion of the linkers within the MIL-140C framework. In Figure S8c three pairs of spinning sidebands are observed. The first sideband to the left exhibits substantially higher intensity in comparison to that to the right, whereas the remaining two pairs of sidebands have comparable intensities. This indicates an axially asymmetric NMR shielding tensor and CSA pattern (δ_*xx*_ ≠ δ_*yy*_ ≠ δ_*zz*_). In the case of rapid linker rotation, the partial averaging of the shielding tensor would occur, and the averaged shielding tensor is expected to display axial symmetry and a narrower CSA pattern.^56,57^ For comparison, the low-temperature asymmetric CSA pattern of benzene collapses into an axially symmetric pattern at 223 K due to rapid molecular reorientation,^58^ which is not the case for the MIL-140C linkers. Furthermore, we calculated the ^13^C δ_aniso_ values of the phenyl groups in MIL-140C linkers on the basis of the structural models determined by cRED. The calculated δ_aniso_ values were in the range of 110–190 ppm with a mean δ_aniso_ value of 162 ppm, which is close to our experimental estimate from MIL-140C of ∼140 ppm. This together with the axially asymmetric CSA pattern suggests the absence of π-flips in MIL-140C and thus confirms that small-amplitude librational motions generated in the potential well around the central position are the origin of the molecular motions in MIL-140C (Figure 4).

**Figure 4:**
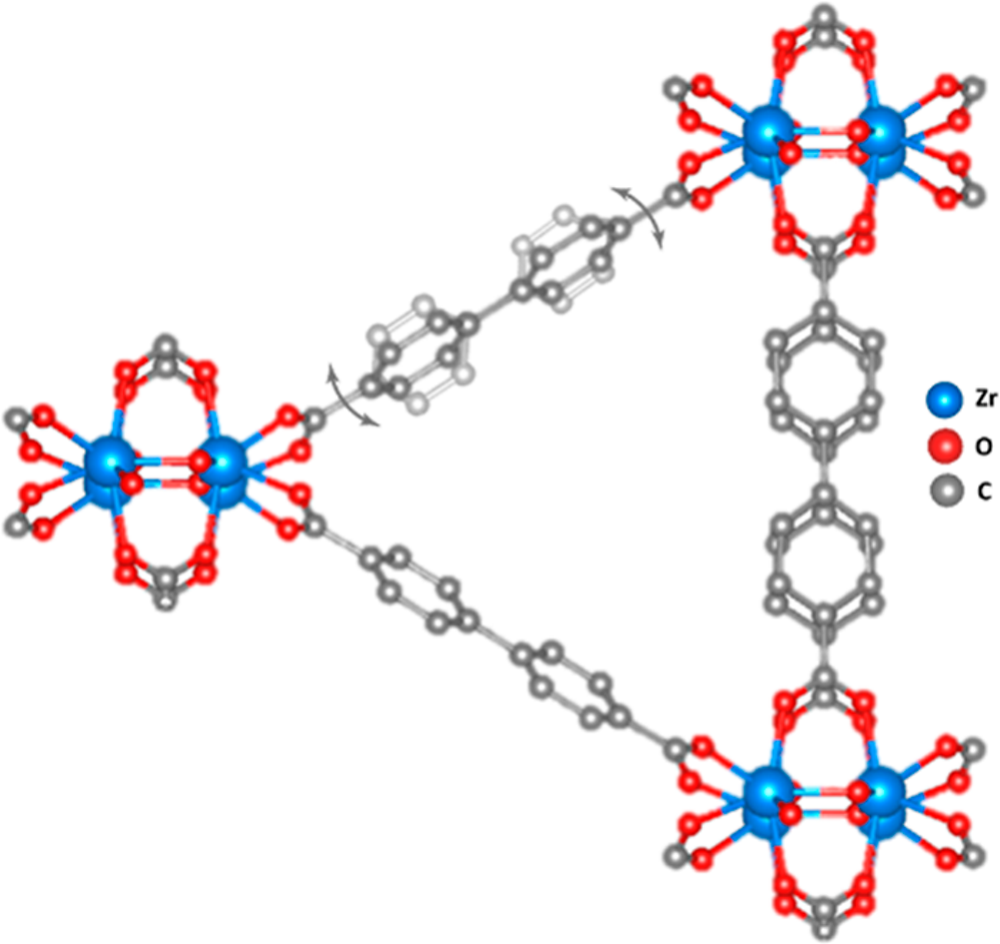
Linker 1 in MIL-140C exhibits small-amplitude librational motions (indicated by the arrows).

In conclusion, we demonstrate that 3D ED can open new opportunities to reveal molecular motions in MOFs. The structural model and dynamic behavior of UiO-67 obtained from cRED data show a good agreement with those by SCXRD and molecular simulation, confirming that 3D ED can achieve high accuracy in structure determination and can probe molecular motions. Importantly, we also demonstrate that 3D ED can be used to distinguish different extents of molecular motions of the same linker in MIL-140C, where motions are identified in the isolated linkers rather than those reinforced by π–π interaction. By providing more details on positional specific motions, it could further open the possibility for tailoring different levels of local dynamics in MOFs. Considering the difficulty in obtaining large crystals and pure samples, we envisage 3D ED to be an important technique in studying molecular motions not only in MOFs but also in other materials, such as covalent organic frameworks and other organic crystals.

## Supporting Information Available

Experimental details, SEM, PXRD, structure refinement, and NMR (PDF)

## Accession Codes

CCDC 2073254–2073255 and 2087354 contain the supplementary crystallographic data for this paper. These data can be obtained free of charge via www.ccdc.cam.ac.uk/data_request/cif, or by emailing data_request@ccdc.cam.ac.uk, or by contacting The Cambridge Crystallographic Data Centre, 12 Union Road, Cambridge CB2 1EZ, UK; fax: +44 1223 336033.
