# Supplementary material for: Probing Molecular Motions in Metal–Organic Frameworks by Three-Dimensional Electron Diffraction
Source: J Am Chem Soc. 2021 Oct 25;143(43):17947–52. doi: 10.1021/jacs.1c08354 (PMC8569804; doi:10.1021/jacs.1c08354)
Supplement: Supplementary file 1 — ja1c08354_si_001.pdf [file ja1c08354_si_001.pdf]

Supplementary Information for

Probing Molecular Motions in Metal-Organic Frameworks by Three-Dimensional Electron Diffraction

Laura Samperisi[†], Aleksander Jaworski[†], Gurpreet Kaur[$], Karl Petter Lillerud[§], Xiaodong Zou[†,*], Zhehao Huang[†,*]

[†]Department of Materials and Environmental Chemistry, Stockholm University, Stockholm SE-106 91, Sweden

[$]Department of Organic Chemistry, Stockholm University, Stockholm SE-106 91, Sweden

[§] Department of Chemistry, Center for Materials Science and Nanotechnology, University of Oslo, P.O. Box 1033, N-0315 Oslo, Norway

Table of Contents:

Section 1. Chemicals and instrumentation

Section 2. Synthesis of UiO-67 and MIL-140C

Section 3. Structural analysis of UiO-67 and MIL-140C

Section 4. NMR analysis

Section 1. Chemicals and Instrumentation

Chemicals

4,4'-biphenyldicarboxylic acid (bpdc) was bought from Fox-Chemicals whereas zirconium(IV) chloride (≥ 99.5 %), benzoic acid (BA, ≥ 99.5 %) and *N,N*-Dimethylformamide (DMF, ≥ 99.8 %) were bought from Merck. All the chemicals were used as purchased, without further purification, except for DMF. DMF was further purified using Mbraun MB SPS-200 solvent purification system to remove water content.

Instrumentation

Transmission electron microscopy (TEM) and continuous rotation electron diffraction (cRED). Sample preparation for electron diffraction experiments was performed by crashing the powder with pestle and mortar and dispersing it in acetone. Three droplets of the resulting dispersion were deposited onto a carbon-coated copper grid. After complete evaporation of the solvent at room temperature, the grid was loaded on a single-tilt tomography holder (tilting range -70° to +70°) for room temperature data collection or on a cryo-transfer holder (tilting range -70° to +70°) for cryogenic temperature (98 K) data collection. The cRED data collection was performed on a 200 kV JEOL JEM-2100 transmission electron microscope equipped with an Amsterdam Scientific Instruments QTPX262k Timepix hybrid pixel detector (512 × 512 pixels, pixel size 55 μm) by using the software *Instamatic*[1]. Images were recorded by a Gatan Orius 833 CCD camera (2048 × 2048 pixels, pixel size 7.4 μm). To protect the crystals from beam damage, data were acquired using a small condenser aperture (c.a. 50 μm) and under electron dose of c.a. 0.01 e^{-} $Å^{-2}$ s^{-1}. To collect cRED data, a crystal was placed in the electron beam and its height was adjusted to the mechanical eucentric height. A selected area (SA) aperture corresponding to a circular area of 1.5 μm in diameter was used to collect cRED data of UiO-67. Due to the crystal overlap of MIL-140C, smaller SA apertures defining areas from 0.50 to 1 μm in diameter were used for data collection. Because MIL-140C has a plate-like morphology, datasets were collected and merged from crystals both laying and standing on the grid to minimize the effect of preferred orientation. We collected cRED datasets from many crystals, among which we used one dataset from UiO-67 and 9 datasets from MIL-140C collected at 298K. In addition, 9 datasets from MIL-140C were collected at 98K to study the influence of temperature. For both UiO-67 and MIL-140C, data were collected using an exposure time of 0.5 s per frame, a camera length of 30 cm, and a goniometer tilt speed of 0.45° s^{-1}. The goniometer was continuously rotating while selected area electron diffraction patterns were simultaneously recorded.

Powder X-ray diffraction (PXRD). Samples were prepared using ~20 mg of the sample on a glass plate XRD sample holder by spreading it evenly and covering with transparent plastic film. The plastic film gives a small signal in the PXRD patterns observed at 2θ = ~22° and 34° as broad peaks. PXRD patterns (Cu Kα radiation, λ = 1.5418 Å, 2θ range = 2-50°) were collected in reflectance Bragg-Brentano geometry with a Bruker D8 Discovery diffractometer equipped with a focusing Ge-monochromator and a Bruker LYNXEYE detector.

Scanning electron microscopy (SEM). SEM images were taken on a Hitachi SU8230 Field Emission Scanning Electron Microscope (FE-SEM).

Solid state nuclear magnetic resonance (NMR). Magic-angle-spinning (MAS) NMR experiments were performed at a magnetic field of 14.1 T (Larmor frequencies of 600.12, and 150.92 for ^{1}H, and ^{13}C, respectively) on a Bruker Avance-III spectrometer. Spectra were acquired using a 1.3 mm probehead and a 60 kHz MAS rate. ^{1}H MAS NMR acquisition involved a use of a rotor-synchronized, double-adiabatic spin-echo sequence with a 90° excitation pulse of 1.25 µs followed by a pair of 50.0 µs tanh/tan short high-power adiabatic pulses (SHAPs) with 5 MHz frequency sweep[2,3]. All pulses operated at the nutation frequency of 200 kHz, and 128 signal transients were acquired using a relaxation delay of 5 s. Cross-polarization (CP) ^{1}H-^{13}C CPMAS NMR spectrum involved Hartmann-Hahn matched radiofrequency fields applied for a contact interval of 1.5 ms and 85 kHz spinal64 proton decoupling. 16384 signal transients were collected using a relaxation delay of 5 s. To observe spinning sideband pattern resulting from ^{13}C NMR chemical shift anisotropy (CSA), an additional ^{1}H-^{13}C CPMAS acquisition was performed with a 7 mm probehead at a 7 kHz MAS rate and using 65 kHz spinal64 proton decoupling. 2048 signal transients were collected using a relaxation delay of 5 s. Chemical shifts were referenced with respect to neat tetramethylsilane (TMS).

Section 2. Synthesis of UiO-67 and MIL-140C

Synthesis of pure UiO-67. UiO-67 ($Zr_6O_4(OH)_4(O_2C(C_6H_4)_2CO_2)_6$) was synthesized using previously reported procedure[4]. 2.0 g of zirconium chloride (1 eq) was added to the mixture of 0.93 mL distilled water (6 eq) and 33.3 mL of DMF (50 eq) at room temperature on a stirring plate. This solution was heated and 3.14 g of BA (3 eq) was added, stirred until completely dissolved and then 2.92 g of bpdc (1 eq) was added. The solution was transferred to a round bottom flask and heated at 130 °C overnight with stirring and a condenser. The resulting product was filtered and washed with hot DMF and acetone, and dried overnight at 150 °C.

Synthesis of mixed phase UiO-67 and MIL-140C.

A systematic study was performed to examine the effect of addition of small amounts of water in the synthesis of UiO-67 using $ZrCl_4$: 4,4'-biphenyldicarboxylic acid (H_2bpdc): benzoic acid: DMF as 1:1:3:50 molar equiv. In this series the reactions with intermediate amount of water revealed a mixed phase of MIL-140C and UiO-67. Three samples were synthesized, namely mixed phase (5 eq), mixed phase (4 eq) and mixed phase (3 eq) where 0.77 mL (5 eq), 0.62 mL (4 eq) and 0.46 mL (3 eq) of water was added, respectively, in the reaction mixture. These experimental procedures were performed the same as described in the above section.

Synthesis of pure MIL-140C. Synthesis of MIL-140C ($ZrO(O_2C_{12}H_8$-$CO_2)$) was done using the reported protocol with modifications[5]. Typically, 1.0 g of zirconium chloride (1 eq) was added to 16.6 mL of DMF (50 eq) in a 50 mL autoclave. 1.57 g of BA (3 eq) was added under stirring, followed by 1.46 g of bpdc (1 eq). The autoclave was sealed and heated overnight at 220 °C. A white product was obtained which was filtered and washed with hot DMF and acetone, and finally dried overnight at 150 °C.

Discussion. Understanding the role of the reagents and their amount are vital as they can affect the reproducibility of the MOFs. In the synthesis of UiO-67, the reagent ratios play important

roles to obtain defect free crystals[4]. In this work, we study the effect of amount of water. The synthesis was conducted by using molar ratio of $ZrCl_4$:H_2bpdc:BA:H_2O:DMF (anhydrous) = 1:1:3:x:50 (x= 0, 3, 4, 5, 6). When anhydrous DMF is used, the addition of water in the synthesis becomes an important role as it is the only source of water. The PXRD patterns of the obtained products show that pure UiO-67 and MIL-140 were obtained using 0 and 6 equivalent of water, respectively, and phase mixtures of UiO-67 and MIL-140C were obtained using 3, 4, and 5 equivalent of water (Figure S1). The additional peaks (marked by asterisk) in the PXRD patterns are attributed to MIL-140C. Moreover, SEM images show octahedral morphology of UiO-67 and plate-like morphology of MIL-140C (Figure S2).

Section 3. Structural analysis of UiO-67 and MIL-140C

Space groups and unit-cell parameters were initially obtained from the software *REDp*[6]. The unit cell parameters of UiO-67 and MIL-140C are determined as a = 26.51Å b=26.30Å c=26.04 Å and α =90.56°, β =90.93° γ = 90.57°, and a = 31.47 Å, b = 15.57 Å, c = 7.99 Å, α = 90.88°, β = 101.26°, and γ = 89.2°, respectively. The parameters are reasonably close to a cubic cell for UiO-67 and a monoclinic cell for MIL-140C. From the two-dimensional (2D) slices cut from the reconstructed 3D reciprocal lattice, the reflection conditions can be deduced as hkl: $h+k$ = 2n, $h+l$ = 2n, $k+l$ = 2n; 0kl: k,l = 2n; hhl: $h+l$ = 2n and 00l: l = 2n for UiO-67 (Figure S3) and hkl: $h+k$ = 2n; h0l: h,l = 2n and 0k0: k = 2n for MIL-140C (Figure S4). Therefore, the possible space groups are $F23$ (No. 196), $Fm\bar{3}$ (No. 202), $F432$ (No. 209), $F4_132$ (No. 210), $F\bar{4}3m$ (No. 216), and $Fm\bar{3}m$ (No. 225) for UiO-67 and $C2$ (No. 5), Cc (No. 9), Cm (No. 10) and $C2/c$ (No. 15) for MIL-140C. The normalized structure factor <| E^2-1 |> statistics is 0.919 and 0.968 for UiO-67 and MIL-140C, respectively, indicating that the structures are centrosymmetric. Thus, the possible space groups for UiO-67 are $Fm\bar{3}$ (No. 202) and $Fm\bar{3}m$ (No. 225), and for MIL-140C is $C2/c$ (No. 15). Between $Fm\bar{3}$ and $Fm\bar{3}m$, we chose the higher symmetry space group for UiO-67.

The data were subsequently processed through *XDS*[7,8] to extract the reflection intensities. The unit cell parameters of UiO-67 and MIL-140C are refined as a = b= c= 26.880(3) Å and α = β = γ = 90°, and a = 32.360(7) Å, b = 15.800(3) Å, c = 7.910(2) Å, α = 90°, β = 103.00(3)° and γ = 90°, respectively. Although some diffuse scattering along the a*-axis is observed (Figure S4c), it is very weak and therefore not considered in the structure determination. The structures were solved *ab initio* with dual-space algorithm implemented in *SHELXT* and refined with a full- matrix least squares technique using *SHELXL*[9,10]. During the refinement, atomic scattering factors and wavelength for electrons were used. For both MOFs, all the non-hydrogen atom positions could be directly located from the initial structure solution. Hydrogen atoms were added with HFIX 43. For UiO-67, the structure was solved and refined from an individual dataset. During the refinement of UiO-67, soft geometrical restrains DANG were applied on the linker. The refinement converged with the agreement values R_1=0.215 for 404 reflections with Fo > 4σ(Fo) and 0.249 for all 703 reflections (Table S1). For MIL-140C, because of the preferred orientation, three datasets per temperature from individual crystals were merged for structure determination. The merging was performed with XSCALE, which is part of the XDS package. The correlation coefficients of the common reflection intensities (CC_I) were used to assess the quality of merging (Table S4)[11]. During the refinement of MIL-140C, soft

geometrical restrains (DFIX, DANG, and FLAT) were applied on the non π-stacked linker molecule. For the data collected at room temperature, SIMU was applied on carbon atoms along the molecular axis of the π-stacked linker. The refinement converged to R_1=0.169 for 2083 reflections with Fo > 4σ(Fo) and 0.210 for all 3079 data for the crystals at room temperature (298 K), and R_1=0.173 for 1892 reflections with Fo > 4σ(Fo) and 0.200 for all 2685 data for the crystals at cryogenic temperature (98 K) (Table S1). At the end of the refinement, the SWAT[12] parameter was applied to all structures to compensate the effects of solvents in the pores. The relatively high R_1 values are mainly caused by dynamical effects that make the intensities deviated from kinematic intensities. For the analysis of the ADPs, the principal mean square atomic displacements U given as an output by *SHELXL* were used. These values correspond to the three eigenvalues λ1, λ2 and λ3. The values from three different merged datasets, each one obtained by merging three datasets from individual crystals, were averaged and standard deviations for the eigenvalues were calculated.

Section 4. NMR analysis

The ^{1}H solid-state NMR spectrum of MIL-140C revealed five proton resonances at 8.2, 7.5, 6.6, 5.9, and 5.4 ppm, which reflects differences in distances to inorganic fragments and distinct linker conformations (Figure S8a). Signal at 1.7 ppm is attributed to water molecules present in the pores of the material[13]. The 1H-13C CPMAS spectrum shows resonances in the range of 120–150 ppm, which originate from carbon atoms in the phenyl rings of the bpdc linkers, whereas those of carboxyl groups give signals in the 170–180 ppm region (Figure S8b). In addition, the ^{1}H-^{13}C CPMAS spectrum at a slow MAS rate of 7 kHz was recorded to allow observation of the ^{13}C chemical shift anisotropy (CSA) pattern (Figure S8c). The estimate of the chemical shift anisotropy (δaniso) as well as the shape of the CSA pattern provide insights into the potential dynamics and motion of the linkers within the MIL-140C framework. Three pairs of spinning sidebands are observed in Figure S8c. The first sideband at c.a. 180 ppm exhibits substantially higher intensity than its counterpart at c.a. 80 ppm, whereas the remaining two pairs of sidebands have comparable intensities. This indicates an axially asymmetric NMR shielding tensor and CSA pattern (δxx≠δyy≠δzz), which is expected for carbon atoms involved in the phenyl group with restricted motion. In the case of π-flips, the partial averaging of the shielding tensor would occur, and the averaged shielding tensor is expected to display axial symmetry and narrower CSA pattern[14,15]. Moreover, since three pairs of sidebands are present, the shift anisotropy in frequency units can be estimated to be around three times the MAS rate, which translates to an average δaniso=~140 ppm on our experimental setup. Because of relatively wide range of isotropic chemical shifts from phenyl rings of MIL-140C linkers, it was not possible to acquire a spectrum at even lower MAS rate in order to provide better accuracy for chemical shift anisotropy determination by involving more sidebands. The estimated average value of δaniso=~140 ppm is moderately lower than δaniso=180 ppm reported for the benzene at 20 K[16]. However, the low-temperature asymmetric CSA pattern of benzene collapses into an axially symmetric one at 223 K due to rapid molecular reorientation[17], which is not the case for the MIL-140C linkers. Furthermore, the 13C chemical shift anisotropies for phenyl groups of the MIL-140C linkers were also calculated on the models derived from our 3D ED structural model. Calculations were performed with the robust DSD-

PBEP86/pcSseg-2 level of theory[18]. Obtained δaniso values were in the range of 110–190 ppm with the mean chemical shift anisotropy of δaniso=162 ppm, which is close to our estimate for the MIL-140C. This together with the axially asymmetric CSA tensor suggests the absence of π-flips, while librations are probably present in the MIL-140C structure at the room temperature.

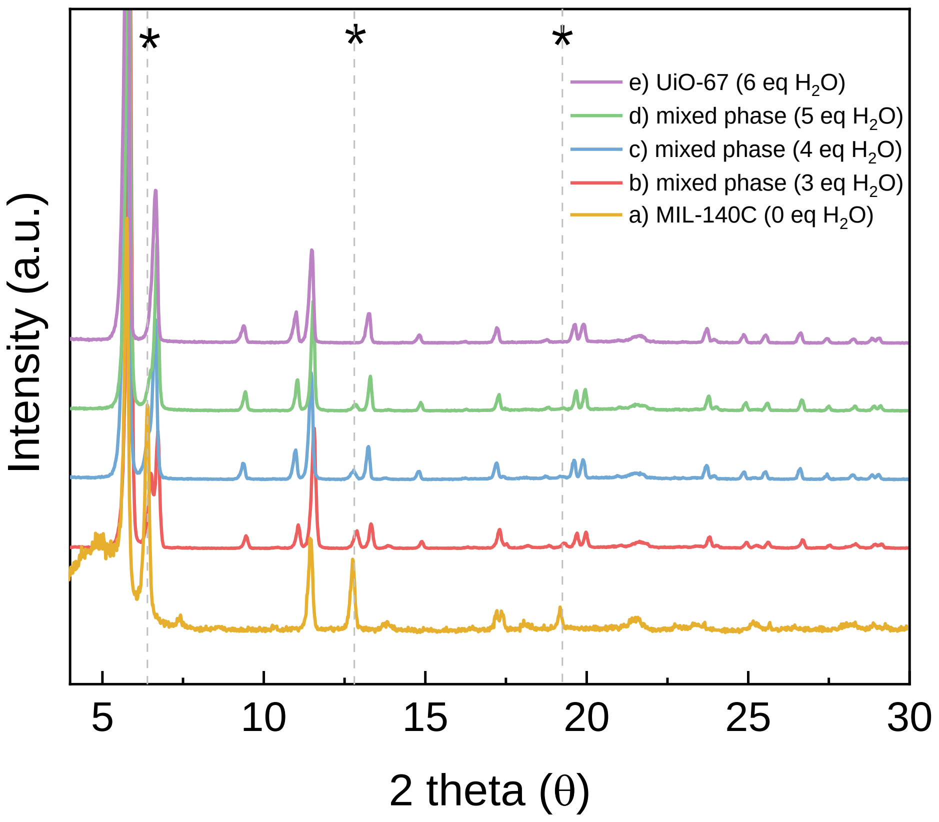

Figure S1. PXRD patterns of (a) MIL-140C, (b-d) mixtures of UiO-67 and MIL-140C, and (e) pure UiO-67. The asterisks indicate the main peaks of MIL-140C.

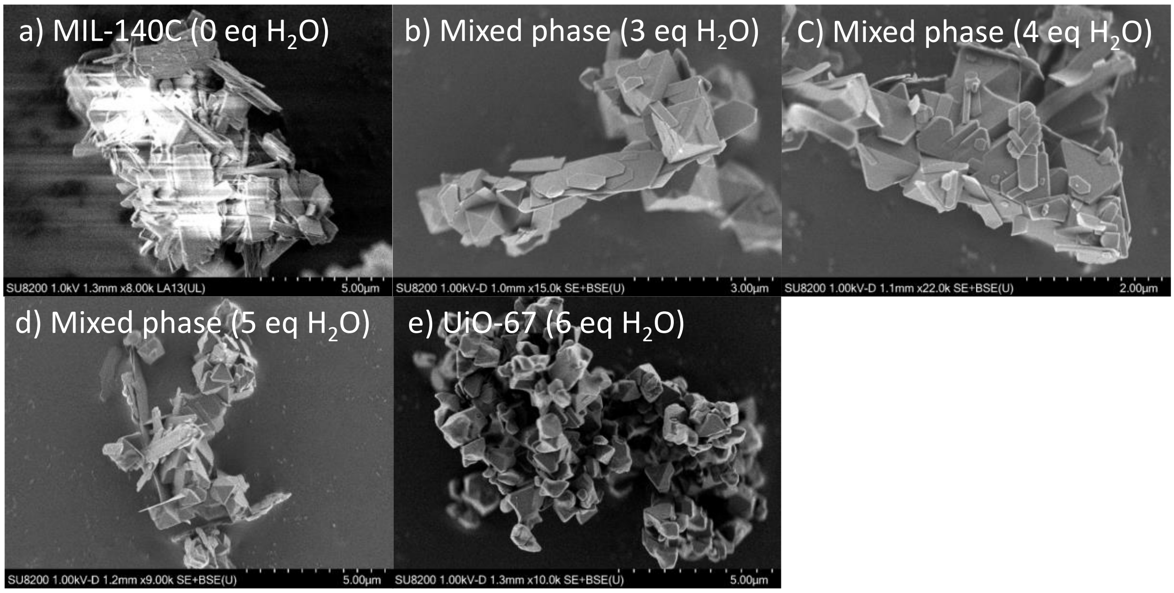

Figure S2. SEM images of samples synthesized using different water contents, (a) pure MIL-140C, (b-d) the mixture of UiO-67 and MIL-140C, and (e) pure UiO-67.

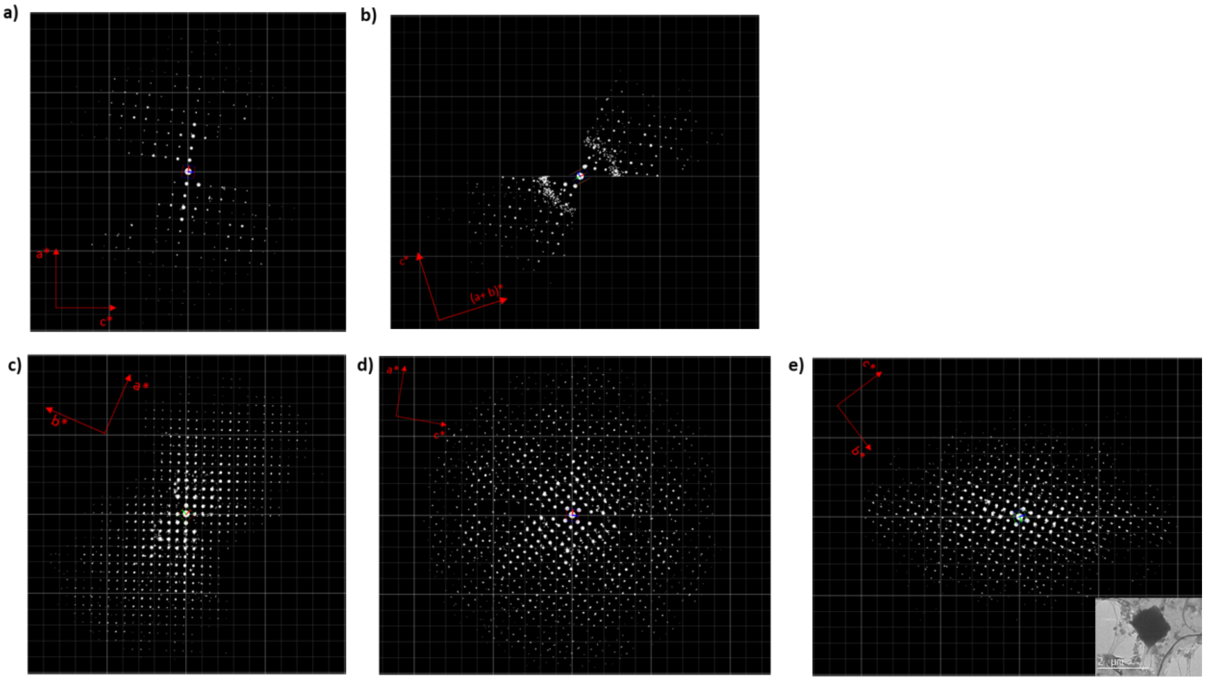

Figure S3. 2D slices from the 3D reconstructed reciprocal lattice of UiO-67 showing the (a) *h*0*l* and (b) *hhl* planes. The 3D reconstructed reciprocal lattice of UiO-67 viewed along the (c) *c**-axis, (d) *b**-axis, and (e) *a**-axis. The inset shows the crystal from which the dataset was collected.

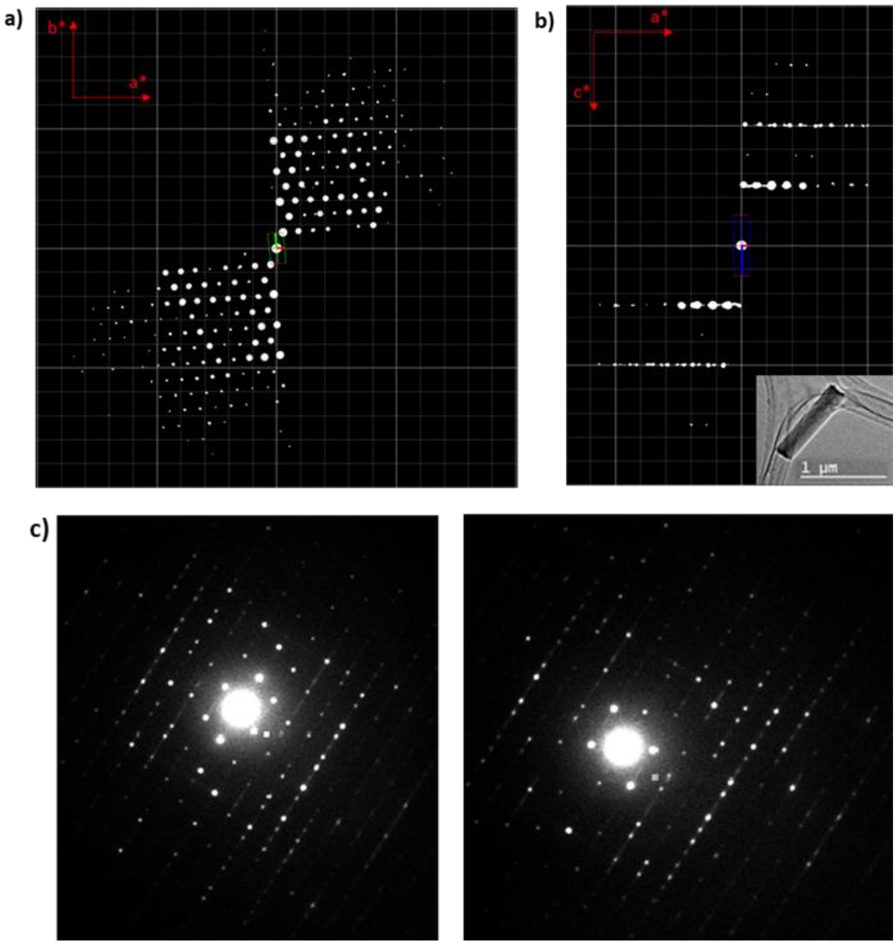

Figure S4. 2D slices cut from the 3D reciprocal lattice reconstructed from the cRED data of MIL-140C showing the (a) *hk*0 and (b) *h*0*l* planes. (c) Individual electron diffraction frames from the cRED data showing weak streaks along *a**-axis, which are also observed in the *h*0*l* plane in (b). The inset in (b) shows the crystal from which the cRED data was collected.

a)

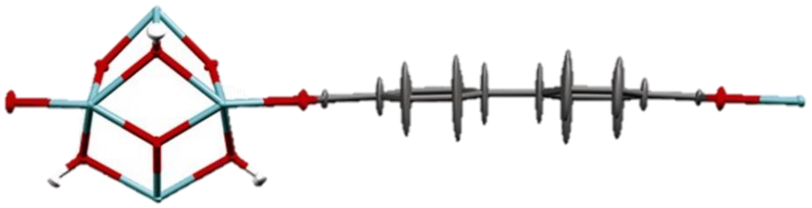

b)

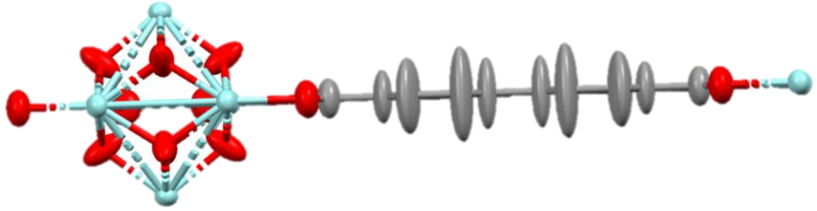

Figure S5. (a) Calculated atomic probability density functions (50 % probability) from molecular dynamics simulation and single crystal X-ray data. Adapted with permission from the work of Hobday and co-workers, ref. 19. Published by John Wiley & Sons, Inc. Copyright under Creative Commons CC BY 4.0, 2016. (b) The thermal ellipsoid model (50% probability) of UiO-67 obtained after refinement against cRED data. The molecular motions in the bpdc linker show a good agreement between those obtained from calculation and cRED data.

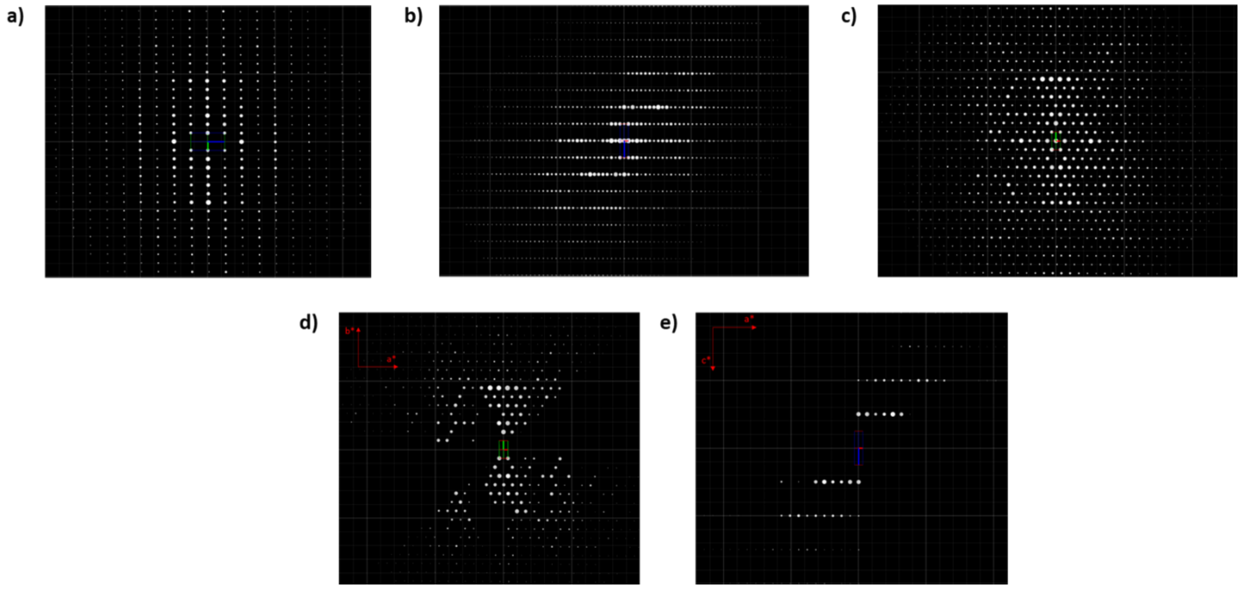

Figure S6. The 3D reconstructed reciprocal lattice of a merged dataset of MIL-140C at 298 K, viewed along the (a) a*-, (b) b*- and (c) c*-axis. 2D slices cut from the reconstructed 3D reciprocal lattice of the same merged dataset showing the (d) *hk*0 and (e) *h*0*l* planes. No *h*00 reflections are present in the data, showing there is a missing cone around the *a**-axis.

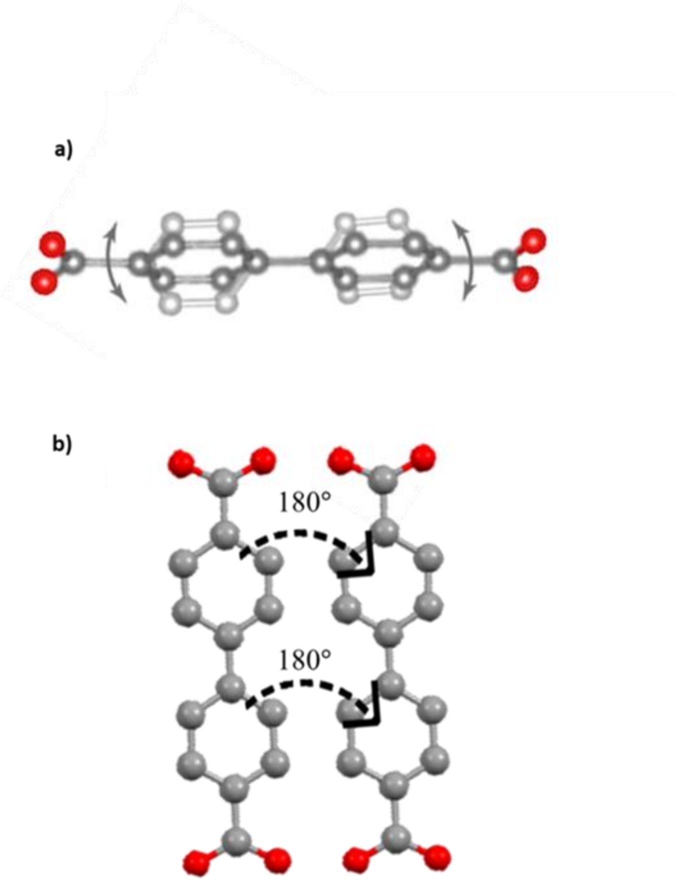

Figure S7. Illustration of molecular motions of (a) libration and (b) π-flip.

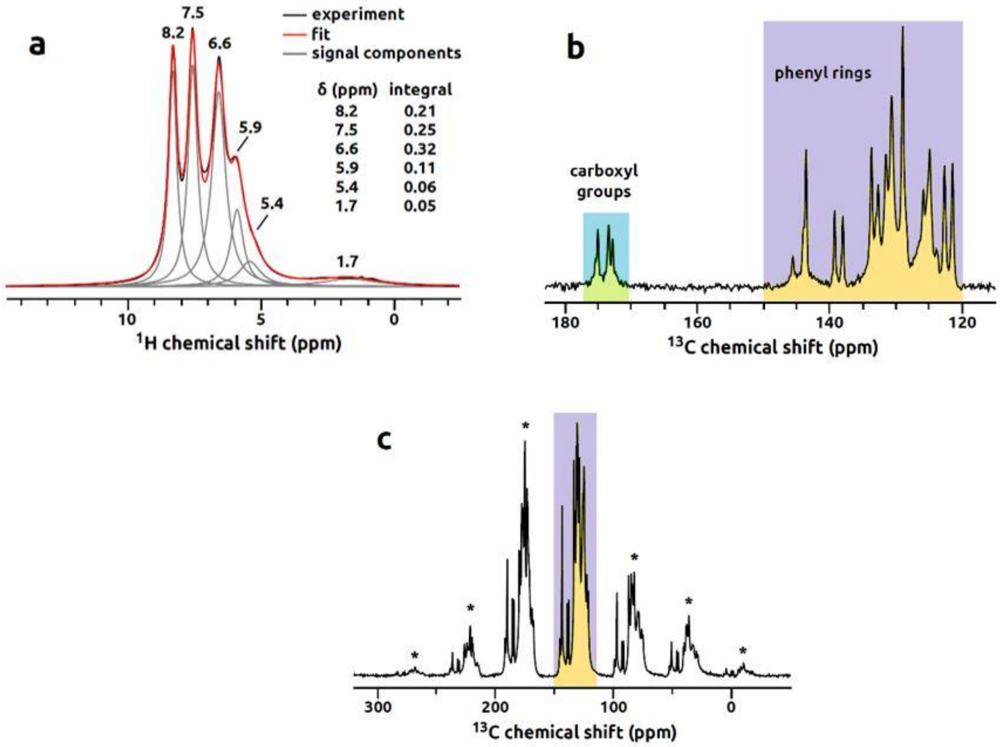

Figure S8. (a) ^{1}H MAS NMR and (b) ^{1}H-^{13}C CPMAS spectra of MIL-140C collected at 14.1 T and the MAS rate of 60.00 kHz. 5% of the total signal integral in ^{1}H MAS NMR is identified belonging to the guest molecules (c) ^{1}H-^{13}C CPMAS spectrum recorded at the MAS rate of 7.00 kHz is shown with spinning sidebands marked with asterisks.

Table S1. Crystallographic details and refinement parameters for UiO-67 and MIL-140C.

	**UiO-67**	**MIL-140C**	**MIL-140C**
Crystal system	Cubic	Monoclinic	Monoclinic
Space group	$Fm\bar{3}m$ (No. 225)	$C2/c$ (No. 15)	$C2/c$ (No. 15)
a (Å)	26.880(3)	32.360(7)	32.670(7)
b (Å)	= a	15.800(3)	15.900(3)
c (Å)	= a	7.910(2)	8.030(2)
α (°)	90	90	90
β (°)	90	103.00(3)	104.200(3)
γ (°)	90	90	90
Temperature (K)	298(2)	298(2)	98(2)
Resolution (Å)	0.90	0.80	0.80
Number of unique reflections	703	3079	2685
Number observed reflections (I > 2 sigma(I))	404	2083	1892
Completeness (%)	100	80.1	68.1
No. refined parameters	40	186	186
No. restraints	6	35	17
R_{int}	0.323	0.244	0.278
R_1 for Fo>4 sigma (Fo)	0.215	0.169	0.173
R_1 for all	0.249	0.210	0.200
Goof	1.460	1.460	1.447

Table S2. Differences of the atomic positions in UiO-67 refined against cRED and SCXRD data[20].

Atoms	Difference of the atomic position (Å)
**Zr1**	0.0101(5)
**O1**[a]	0.251(5) / 0.362(5)
**O2**	0.0159(8)
**C1**	0.021(1)
**C2**	0.018(1)
**C3**[b]	0.34(4)
**C4**[b]	0.32(3)
**C5**	0.025(1)

[a]In the structural model obtained by SCXRD, O1 was split and modeled as disordered. We compared results with and without splitting. Because of the disorder, a large deviation is generated.
[b]The large deviation is a result of the linker motion.

Table S3. Refinement details of merged datasets of MIL-140C.

**Dataset no.**	**1**	**2**	**3**	**4**	**5**	**6**
Temperature (K)	298	298	298	98	98	98
Resolution (Å)	0.80	0.80	0.80	0.80	0.80	0.80
No. of unique reflections	3079	2555	2933	2685	2690	2619
No. observed reflections ($I > 2$ sigma(I))	2083	1715	2067	1892	1997	1954
Completeness (%)	80.1	76.7	76.9	68.1	66.0	64.3
No. refined parameters	186	180	186	186	186	186
No. restraints	35	22	22	17	22	22
R_{int}	0.244	0.223	0.212	0.278	0.307	0.273
R_1 for Fo>4 sigma (Fo)	0.169	0.161	0.164	0.173	0.180	0.185
R_1 for all	0.210	0.198	0.206	0.200	0.206	0.214
Goof	1.460	1.331	1.574	1.447	1.520	1.568

Table S4. The correlation coefficients of the common reflection intensities (CC_I) between each two datasets.

**Merged dataset no.**	**Temperature (K)**	**Individual Dataset no.**	**Individual Dataset no.**	**No. of common reflections**	**CC_I**
		1	2	920	0.961
1	298	1	3	1094	0.966
		2	3	873	0.948
		1	2	994	0.939
2	298	1	3	880	0.959
		2	3	981	0.902
		1	2	1194	0.975
3	298	1	3	1319	0.938
		2	3	1592	0.944
		1	2	893	0.966
4	98	1	3	871	0.985
		2	3	1154	0.967
		1	2	1441	0.903
5	98	1	3	1154	0.967
		2	3	1713	0.910
		1	2	871	0.966
6	98	1	3	1390	0.967
		2	3	1713	0.910

References

(1) Cichocka, M. O.; Ångström, J.; Wang, B.; Zou, X.; Smeets, S. High-Throughput Continuous Rotation Electron Diffraction Data Acquisition via Software Automation. *J. Appl. Crystallogr.* **2018**, *51* (6), 1652–1661.

(2) Hwang, T.-L.; van Zijl, P. C. M.; Garwood, M. Fast Broadband Inversion by Adiabatic Pulses. *J. Magn. Reson.* **1998**, *133* (1), 200–203.

(3) Kervern, G.; Pintacuda, G.; Emsley, L. Fast Adiabatic Pulses for Solid-State NMR of Paramagnetic Systems. *Chem. Phys. Lett.* **2007**, *435* (1), 157–162.

(4) Kaur, G.; Øien-Ødegaard, S.; Lazzarini, A.; Chavan, S. M.; Bordiga, S.; Lillerud, K. P.; Olsbye, U. Controlling the Synthesis of Metal–Organic Framework UiO-67 by Tuning Its Kinetic Driving Force. *Cryst. Growth Des.* **2019**, *19* (8), 4246–4251.

(5) Butova, V. V.; Budnyk, A. P.; Charykov, K. M.; Vetlitsyna-Novikova, K. S.; Lamberti, C.; Soldatov, A. V. Water as a Structure-Driving Agent between the UiO-66 and MIL-140A Metal–Organic Frameworks. *Chem. Commun.* **2019**, *55* (7), 901–904.

(6) Wan, W.; Sun, J.; Su, J.; Hovmöller, S.; Zou, X. Three-Dimensional Rotation Electron Diffraction: Software RED for Automated Data Collection and Data Processing. *J. Appl. Crystallogr.* **2013**, *46* (6), 1863–1873.

(7) Kabsch, W. XDS. *Acta Crystallogr. D Biol. Crystallogr.* **2010**, *66* (2), 125–132.

(8) Kabsch, W. Integration, Scaling, Space-Group Assignment and Post-Refinement. *Acta Crystallogr. D Biol. Crystallogr.* **2010**, *66* (Pt 2), 133–144.

(9) Sheldrick, G. M. A Short History of SHELX. *Acta Crystallogr. A* **2008**, *64* (1), 112–122.

(10) Sheldrick, G. M. SHELXT – Integrated Space-Group and Crystal-Structure Determination. *ActaCrystallogr. Sect. Found. Adv.* **2015**, *71* (1), 3–8.

(11) Ge, M.; Yang, T.; Wang, Y.; Carraro, F.; Liang, W.; Doonan, C.; Falcaro, P.; Zheng, H.; Zou, X.; Huang, Z. On the Completeness of Three-Dimensional Electron Diffraction Data for Structural Analysis of Metal- Organic Frameworks. Faraday Discuss. **2021**, DOI: 10.1039/d1fd00020a.

(12) R. Langridge, D.A. Marvin, W.E. Seeds, H.R. Wilson, C.W. Hooper, M.H.F. Wilkins and L.D. Hamilton, J. Mol. Biol. **1960** (2), 38-64.

(13) Leubner, S.; Siegel, R.; Franke, J.; Wharmby, M.T.; Krebs, C.; Reinsch, H.; Senker, J.; Stock, N.; Design and Precursor-based Solid-State Synthesis of Mixed-Linker Zr-MIL-140A. *Inorg. Chem*. **2020** 59 (20), 15250-15261.

(14) Gall, C. M.; Cross, T. A.; DiVerdi, J. A.; Opella, S. J. Protein Dynamics by Solid-State NMR: Aromatic Rings of the Coat Protein in Fd Bacteriophage. *Proc. Natl. Acad. Sci. U. S. A.* **1982**, *79* (1), 101–105.

(15) Duncan, T. M. 13C Chemical Shieldings in Solids. *J. Phys. Chem. Ref. Data* **1987**, *16* (1), 125–151.

(16) Strub, H.; Beeler, A. J.; Grant, D. M.; Michl, J.; Cutts, P. W.; Zilm, K. W. Low-Temperature Carbon-13 Magnetic Resonance in Solids. 2. Six .Pi.-Electron Ring Systems: Tropylium Cation (C7H7+), Benzene (C6H6) and Cyclopentadienide Anion (C5H5-). *J. Am. Chem. Soc.* **1983**, *105* (10), 3333–3334.

(17) Pines, A.; Gibby, M. G.; Waugh, J. S. Proton-Enhanced Nuclear Induction Spectroscopy 13C Chemical Shielding Anisotropy in Some Organic Solids. *Chem. Phys. Lett.* **1972**, *15* (3), 373–376.(16) Stoychev, G. L.; Auer, A. A.; Neese, F. Efficient and Accurate Prediction of Nuclear Magnetic Resonance Shielding Tensors with Double-Hybrid Density Functional Theory. *J. Chem. Theory Comput.* **2018**, *14* (9), 4756–4771.

(18) Rzepka, P.; Bacsik, Z.; Pell, A. J.; Hedin, N.; Jaworski, A. Nature of Chemisorbed CO2 in Zeolite A. *J. Phys. Chem. C* **2019**, *123* (35), 21497–21503.

(19) Hobday, C. L.; Marshall, R. J.; Murphie, C. F.; Sotelo, J.; Richards, T.; Allan, D. R.; Düren, T.; Coudert, F.-X.; Forgan, R. S.; Morrison, C. A.; Moggach, S. A.; Bennett, T. D. A Computational and Experimental Approach Linking Disorder, High-Pressure Behavior, and Mechanical Properties in UiO Frameworks. *Angew. Chem. Int. Ed.* **2016**, *55* (7), 2401–2405.

(20) Øien, S.; Wragg, D.; Reinsch, H.; Svelle, S.; Bordiga, S.; Lamberti, C.; Lillerud, K. P. Detailed Structure Analysis of Atomic Positions and Defects in Zirconium Metal–Organic Frameworks. *Cryst. Growth Des.* **2014**, *14* (11), 5370–5372.
